# LINC01089 in cancer: multifunctional roles and therapeutic implications

**DOI:** 10.1186/s12967-024-05693-8

**Published:** 2024-09-27

**Authors:** Qiang Yi, Gangfeng Zhu, Xinting Ouyang, Weijian Zhu, Kui Zhong, Zheng Chen, Jinghua Zhong

**Affiliations:** 1 The First Clinical Medical College, Gannan Medical University, Ganzhou, 341000 Jiangxi China; 2 Department of Oncology, The First Affiliated Hospital of Gannan Medical University, 128 Jinling Road, Ganzhou, 341000 Jiangxi China

**Keywords:** LncRNA, LINC01089, Malignant cancers, Biological, Clinical therapies

## Abstract

LINC01089 is a prime example of a long non-coding RNA that plays a pivotal role in the progression of human cancers. The gene encoding this lncRNA is located on 12q24.31. LINC01089 has been demonstrated to exert tumor-suppressive effects in various cancers, including colorectal cancer, gastric cancer, lung cancer, ovarian cancer, cervical cancer, papillary thyroid carcinoma, breast cancer, and osteosarcoma. However, its role in hepatocellular carcinoma shows significant discrepancies across different studies. In this review, we systematically explore the functions of LINC01089 in human cancers through bioinformatics analysis, clinical studies, animal models, and fundamental experimental research. Furthermore, we delve into the biological mechanisms and functions of LINC01089, and discuss its potential as a future biomarker and therapeutic target in detail.

## Introduction

Cancer represents a major public health challenge globally, with nearly 10 million deaths annually attributed to the disease [1, 2]. The intricate pathophysiology of cancer arises from a multitude of factors, including genetic dysregulation, epigenetic modifications, and environmental influences [3–5]. Due to individual variations, tumor heterogeneity, and inherent limitations of current treatments, prognosis remains poor for the majority of patients [6]. Consequently, the identification of novel therapeutic targets is imperative. Understanding the intricate networks underpinning tumorigenesis and cancer progression is crucial for advancing cancer diagnosis and therapy. Recent research has unveiled key genetic alterations and dysregulations associated with oncogenesis, paving the way for precision molecular medicine. Long non-coding RNAs (lncRNAs) have emerged as promising biomarkers with significant potential applications.

Long non-coding RNAs are transcripts exceeding 200 nucleotides in length that lack an open reading frame for protein coding [7]. Typically transcribed by RNA polymerase II, they undergo splicing and polyadenylation and regulate gene expression through various pathways and molecular mechanisms, participating in diverse biological processes [8]. Acting as “RNA sponges” or competing endogenous RNAs (ceRNAs), they modulate tumor-related biological processes, including cell proliferation, metastasis, and drug resistance [9]. Their roles encompass sequestering microRNAs, binding to RNA-binding proteins, regulating gene transcription, influencing alternative splicing, and affecting protein translation [10]. Dysregulation of lncRNAs is linked to numerous diseases, such as cardiovascular diseases [11], neurodegenerative diseases [12], metabolic disorders [13], autoimmune diseases [14], infectious diseases [15] and inflammatory conditions [16]. By impacting gene expression and cellular processes, lncRNAs contribute to both normal physiological functions and disease pathogenesis [17]. Therefore, elucidating the molecular mechanisms and pathogenic roles of lncRNAs in human diseases is of paramount importance.

*LINC01089*, also known as *LIMT* (*Long Non Coding RNA Inhibiting Metastasis*), is a 7680-nucleotide transcript located on chromosome 12q24.31 (https://www.ncbi.nlm.nih.gov/gene/338799). It was first identified by Labonne et al. in 2016 during research on microdeletion syndromes [18]. Subsequent studies have shown that this gene is inhibited by EGF in breast cancer, promoting extracellular matrix invasion in vitro and tumor metastasis in vivo [19]. RNAseq and microarray data indicate that this lncRNA is expressed in various immune, neural, muscular, visceral, secretory, and reproductive tissues (https://www.genecards.org/cgi-bin/carddisp.pl?gene=LINC01089&keywords=LIMT). Additionally, substantial evidence suggests that LINC01089 exhibits aberrant expression levels in multiple human cancers, such as hepatocellular carcinoma, lung cancer, and gastric tumors. In recent years, extensive research has been conducted to elucidate the role of this gene in various diseases, including different cancer types, highlighting its significant involvement in disease progression.

This review comprehensively examines the latest findings on the role of LINC01089 across a spectrum of human cancers, delving into its expression patterns and molecular mechanisms in different cancer types, and evaluating its feasibility as a prognostic and diagnostic biomarker. The aim of this review is to deepen our understanding of the multifaceted roles of LINC01089 in oncology and underscore its potential as a pivotal biomarker and therapeutic target.

## Genetic information for LINC01089

**Fig. 1:**
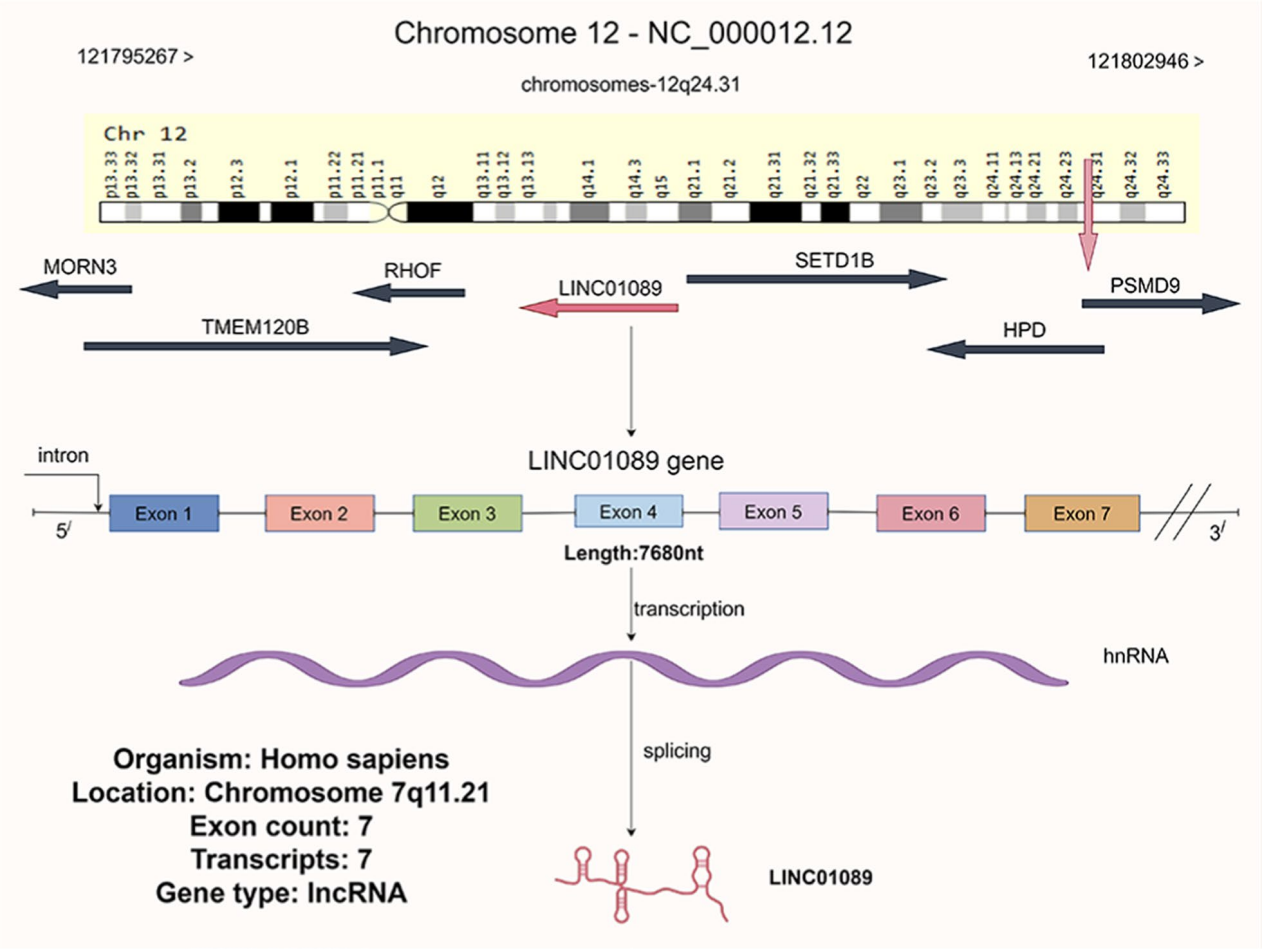
LINC01089 is located on human chromosome 12 and comprises 7 exons, with the potential to generate up to 7 transcript variants according to the NCBI database (the chromosome map is provided by the Genecard website, while the rest is created using the Figdraw 2.0 platform)

The *LINC01089* gene is transcribed from chromosome 12q24.31, spanning 7680 nucleotides of genomic DNA. It is situated between the *SETD1B* and *RHOF* genes, with respective distances of 1063 bp and 1579 bp. In the latest GRCh38 assembly, *LINC01089* (NC_000012.12) is positioned on chromosome 12 from nucleotide 121,795,267 to 121,802,946, encompassing a total of 7680 nucleotides. LINC01089 comprises seven exons and six introns, yielding seven transcript variants (https://www.ncbi.nlm.nih.gov/gene/338799) (as shown in Fig. 1). Multiple promoter or enhancer sites for the *LINC01089* gene have been documented (https://www.genecards.org/), with five transcription factor promoter sites identified as solitary nucleotide transcriptional regulatory elements: GH12J121783, GH12J122020, GH12J122265, GH12J121209, and GH12J121887, all of which can function as promoter or enhancer sites. These five sites are located within 600 kb of the transcription start site and can bind to 145 to over 379 transcription factors.

## Bioinformatics study of LINC01089

**Fig. 2:**
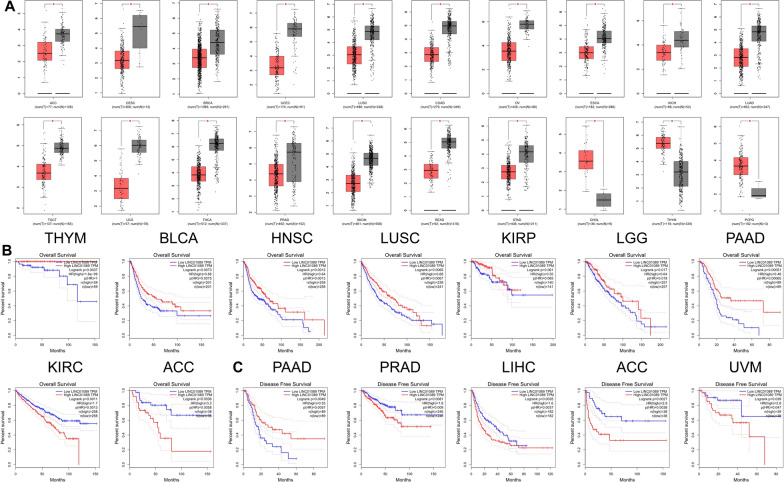
Bioinformatic analysis of LINC01089 in different cancer types (http://gepia.cancer-pku.cn/detail.php?gene=LINC01089). **A** demonstrated the expression levels of LINC01089 in different cancer types. **B** demonstrated the Kaplan–Meier survival curves of overall survival (OS) of LINC01089 in different cancer types. **C** demonstrated the Kaplan–Meier survival curves of progression-free survival (PFS) of LINC01089 in different cancer types. *ACC* adrenocortical carcinoma, *CESC* cervical squamous cell carcinoma and endocervical adenocarcinoma, *BRCA* breast invasive carcinoma, *UCEC* uterine corpus endometrial carcinoma, *LUSC* lung squamous cell carcinoma, *COAD* colon adenocarcinoma, *OV* ovarian serous cystadenocarcinoma, *ESCA* esophageal carcinoma, *KICH* kidney chromophobe, *LUAD* lung adenocarcinoma, *UCS* uterine carcinosarcoma, *THCA* thyroid carcinoma, *PRAD* prostate adenocarcinoma, *SKCM* skin cutaneous melanoma, *READ* rectum adenocarcinoma, *STAD* stomach adenocarcinoma, *CHOL* cholangiocarcinoma, *THYM* thymoma, *PCPG* pheochromocytoma and paraganglioma, *BLCA* bladder urothelial carcinoma, *HNSC* head and neck squamous cell carcinoma, *KIRP* kidney renal papillary cell carcinoma, *LGG* lower grade glioma, *PAAD* pancreatic adenocarcinoma, *KIRC* kidney renal clear cell carcinoma, *LIHC* liver hepatocellular carcinoma, *UVM* uveal melanoma

The expression levels of LINC01089 exhibit significant variation across 20 different cancer types. For instance, in cancers such as cholangiocarcinoma (CHOL), thymoma (THYM), and pheochromocytoma and paraganglioma (PCPG), LINC01089 expression is notably high, whereas in other cancers, it is comparatively low. Kaplan–Meier survival curves demonstrate a significant correlation between LINC01089 expression levels and overall survival (OS) and progression-free survival (PFS) in various cancer types. For example, in clear cell renal cell carcinoma (KIRC), adrenocortical carcinoma (ACC), prostate adenocarcinoma (PRAD), hepatocellular carcinoma (LIHC), and uveal melanoma (UVM), patients with high expression of LINC01089 have markedly poorer survival rates compared to those with low expression. Conversely, the opposite trend is observed in other cancers (as shown in Fig. 2). This indicates that LINC01089 expression may influence cancer progression and patient prognosis. The differential expression suggests that LINC01089 may have distinct biological functions across various cancer types. Given its significant association with patient prognosis in multiple cancers, LINC01089 holds potential as a diagnostic or prognostic biomarker. By assessing LINC01089 expression levels, it may be possible to predict patient outcomes and potentially guide personalized treatment strategies. Collectively, these findings underscore the pivotal role of LINC01089 in cancer biology and its potential as a diagnostic and prognostic marker.

## Clinical sample study of LINC01089

Long non-coding RNAs (lncRNAs), a class of transcripts that do not encode proteins, have garnered increasing attention for their role in various diseases. LINC01089 exhibits significantly dysregulated expression in several diseases, including hepatocellular carcinoma, lung cancer, gastric cancer, and breast cancer, and is associated with various clinical features. Expression analyses reveal that LINC01089 is downregulated in nearly all types of malignant tissues, with the exception of hepatocellular carcinoma (as shown in Table 1). For instance, LINC01089 is lowly expressed in lung adenocarcinoma tissues, correlating with larger tumor size and poorer histological differentiation [20]. Similarly, low levels of LINC01089 in gastric cancer patients are associated with lymphatic metastasis. Moreover, LINC01089 expression is linked to vascular invasion and TNM staging [21, 22]. Studies have also demonstrated that LINC01089 is significantly downregulated in breast cancer specimens and is associated with estrogen receptor-positive status [23]. Intriguingly, in hepatocellular carcinoma tissues, LINC01089 expression has been reported to be both downregulated and upregulated in different studies, with upregulation associated with poor patient prognosis [24, 25]. This suggests that LINC01089 may have a multifaceted role in hepatocellular carcinoma, acting as a tumor suppressor in some contexts while promoting tumor progression in others.

**Table 1:** The Clinicopathological characteristics of LINC01089 in various malignancies

Cancer type	Number of cases	Expression	Clinicopathological characteristics	Prognosis	Refs.
Hepatocellular carcinoma (HCC)	89	High	Poor prognosis	Poor	[24]
	33	Low	Large tumor size, TNM stage, HBsAg	Poor	[25]
	45	Low	–	Poor	[26]
Lung adenocarcinoma (LUAD)	67	Low	Larger tumor size, histological differentiation	Poor	[20]
Gastric cancer (GC)	87	Low	Larger tumor size, T stage, as well as lymphatic metastasis	Poor	[21]
	80	Low	T stage, N stage, TNM stage and vascular invasion	Poor	[22]
Cervical cancer (CC)	60	Low	Larger tumor size and positive lymph node metastasis	Poor	[27]
Small cell lung cancer(SCLC)	60	Low	TNM classification, lymph node metastasis, and poor differentiation	Poor	[28]
Colorectal cancer (CRC)	57	Low	–	Poor	[29]
Breast cancer (BC)	63	Low	Age, lymph node metastasis, TNM stage, ER (estrogen receptor)	Poor	[23]
Glioma	127	Low	WHO classification grading scale, the Karnofsky Perfor-mance Status (KPS) and poor prognosis	Poor	[30]

## Animal studies of LINC01089

The oncogenic role of LINC01089 has been validated in various xenograft models of different cancers in Table 2. Reports indicate that downregulation of LINC01089 can enhance metastasis and in vivo growth of lung cancer. Conversely, upregulation of LINC01089 exerts an opposite effect in ovarian cancer and osteosarcoma. However, xenograft models of hepatocellular carcinoma have yielded disparate results regarding LINC01089's function.

**Table 2:** Effects of LINC01089 on growth and metastasis of cancer xenografts

Cancer type	Animal models	Function	Refs.
Hepatocellular carcinoma	5-week-old male nude BALB/c mice	↑↑ LINC01089: ↑liver metastasis of HCC cells	[24]
	4–5-weeks-old Female BALB/c nu/nu mice	↓↓ LINC01089: ↑ tumor growth	[25]
	6-week-old male BALB/c-nu nude mice	↑↑ LINC01089: ↓tumor sizes, tumor metastasis	[26]
Lung adenocarcinoma	4-week-old BALB/c nudemice	↓↓ LINC01089: ↑ tumor sizes, tumor metastasis, the number of metastatic nodules, Ki-67	[31]
Osteosarcoma	4-week-old BALB⁄c athymic nude mice	↑↑ LINC01089: ↓tumor sizes, Ki67,MMP2, MMP9 and MMP14↑cleaved caspase-3	[32]
Small cell lung cancer	4–6-weeks-old female BALB/c nude mice	↓↓ LINC01089: ↑ tumor sizes, tumor metastasis, chemoresistance	[33]
Non-small cell lung cancer	BALB/c athymic nude mice	↓↓ LINC01089: ↑ tumor sizes, tumor metastasis	[28]
Ovarian cancer	8–12-weeks- old Female athymic nude mice	↑↑ LINC01089: ↓ascites formation, tumor nodules and tumor weight	[34]
Glioma	SPF male BALB/c nude mice	↑↑ LINC01089: ↓tumor growth and tumor volumes	[30]

↑↑ LINC01089: ↑↑ means LINC01089 overexpression; ↑: ↑ means promoting

↓↓ LINC01089: ↓↓ means LINC01089 knockdown or knockout; ↓: ↓ means inhibiting

Animal studies and in vitro research consistently confirm the largely tumor-suppressive role of LINC01089. In lung cancer animal models, silencing LINC01089 results in increased tumor size, metastasis, a higher number of metastatic nodules, and elevated levels of the proliferation marker Ki-67 [31]. Similarly, experiments in osteosarcoma animal models demonstrate the tumor growth and metastasis-inhibiting effects of LINC01089 [32]. Moreover, silencing LINC01089 not only promotes metastasis but also enhances chemotherapy resistance in non-small cell lung cancer [33]. In ovarian cancer research, LINC01089 has been implicated in ascites formation [34]. Interestingly, in different hepatocellular carcinoma animal models, overexpression of LINC01089 has shown contradictory effects: it both attenuates tumorigenesis and metastasis and promotes cancer cell metastasis [24, 26]. This paradoxical phenomenon suggests that LINC01089 may be regulated by multiple complex factors in different liver cancer contexts and experimental conditions, indicating a multifaceted mechanism of action. Overall, the occurrence and progression of most cancer types are closely associated with LINC01089.

## Biological role of LINC01089 in cancer

LINC01089 plays a crucial role in tumorigenesis and cancer progression. Silencing its expression enhances tumor cell proliferation, invasion, migration, and EMT, while reducing apoptosis and modulating the cell cycle (as shown in Table 3). Additionally, knocking down LINC01089 decreases drug sensitivity during chemotherapy, thereby affecting treatment efficacy. These effects underscore the potential of LINC01089 as a therapeutic target in cancer treatment.

**Table 3:** Biological role of LINC01089 in various cancers

Cancer	Assessed cell lines	Expression	Functional	Related gene	Role	Refs.
Hepatocellular carcinoma	HepG2, SK-Hep-1, Huh7, SNU387 and SNU182	Upregulated	Proliferation, migration, cell cycle and invasion, EMT	E2F1/LINC01089/DIAPH3/ERK/ELK	Oncogene	[24]
	Huh7, Hep3B, and SNU449	Downregulated	Sorafenib resistance, EMT, induced apoptosis	miR-665	Tumor Suppressor Gene	[25]
	Huh-7 and LM3	Downregulated	Proliferation, migration and invasion	EGF/JAK/STAT3	Tumor Suppressor Gene	[26]
Lung adenocarcinoma	H1299, CALU-1, HOP62, SPC-A-1, and A549	Downregulated	Proliferation, migration and invasion	YY1/LINC01089/miR-301b-3p/HPGD/AKT/STAT	Tumor Suppressor Gene	[31]
	PC9, H2073, H-1975 and A549	Downregulated	Proliferation, migration and apoptosis	miR-301b-3p/STARD13	Tumor Suppressor Gene	[35]
	A549, Calu3, and HCC827	Downregulated	Proliferation, apoptosis and cell cycle	miR-543/BMP2 and ADCY6	Tumor Suppressor Gene	[20]
Gastric cancer	AGS, BGC-823, HGC-27, MGC-803, SGC-7901	Downregulated	Proliferation, migration and invasion	miR-27a-3p/TEF1	Tumor Suppressor Gene	[21]
	AGS, BGC-823, HGC-27, MGC-803, MKN45	Downregulated	Proliferation and EMT	miR-27a-3p	Tumor Suppressor Gene	[22]
Thyroid cancer	TPC-1 and CAL-62	Downregulated	Migration and invasion	miR-27b-3p/FBLN5	Tumor Suppressor Gene	[36]
Osteosarcoma	U2OS, Saos-2, HOS and 143B	Downregulated	Proliferation, migration, invasion and apoptosis	Hippo pathway	Tumor Suppressor Gene	[32]
Cervical cancer	SiHa, Caski, HeLa, and C4-1	Downregulated	Proliferation and migration	miR-27a-3p/BTG2	Tumor Suppressor Gene	[27]
Small cell lung cancer	H69, H69AR, H446, H460 and H520	Downregulated	Proliferation, migration and chemoresistance	FAK–ERK–REST axis	Tumor Suppressor Gene	[33]
Non-small cell lung cancer	A549 and H1299	Downregulated	Cell cycle and EMT	miR-27a/SFRP1/Wnt/beta-catenin	Tumor Suppressor Gene	[28]
	A549 and SK-MES-1	Downregulated	Proliferation, migration and invasion	miR-3187-3p	Tumor Suppressor Gene	[37]
Colorectal cancer	SW480, HT29, SW620, HCT116, as well as LoVo	Downregulated	Proliferation and migration	miR-27b-3p/HOXA10	Tumor Suppressor Gene	[29]
Breast cancer	MDA-MB-231, BT-549, SUM-159, MDA-MB-468, SK-BR-3, MCF-7, YCCB1, and T47D	Downregulated	Migration, invasion and apoptosis	EGF/LINC01089/Wnt/beta-catenin	Tumor Suppressor Gene	[23]
	MCF10A and MDA-MB-231	Downregulated	Migration and invasion	EGF/LINC01089	Tumor Suppressor Gene	[19]
Ovarian cancer	OV90 and OVCA429	Downregulated	Proliferation, migration and invasion, EMT	EGF/ERK/LINC01089	Tumor Suppressor Gene	[34]
Glioma	U251	Downregulated	Proliferation, migration, invasion and apoptosis	–	Tumor Suppressor Gene	[30]

### LINC01089 in tumor cell cycle regulation

LINC01089 has been shown to regulate the cell cycle during tumor development. For instance, Sun et al. [25] found that in hepatocellular carcinoma (HCC) cells treated with sorafenib, the combination of LINC01089 knockdown and sorafenib treatment resulted in lower apoptosis rates and reduced G2/S phase cell proportions compared to cells treated with sorafenib alone. This suggests that LINC01089 may enhance the efficacy of sorafenib treatment by promoting apoptosis and cell cycle arrest. Additionally, other studies have found that overexpression of LINC01089 leads to a significant increase in the proportion of cells in the G1 phase and a decrease in the G2/M phase, causing cell cycle arrest [20, 28, 34]. Further studies in breast cancer have shown that knocking down LINC01089 promotes the progression of cells from the G1 phase to the S phase by upregulating CDK4 and CDK6 expression, thus shortening the cell cycle [23]. In summary, the silencing of LINC01089 can significantly regulate the cell cycle, potentially leading to poor patient prognosis.

### LINC01089’s impact on tumor cell apoptosis and proliferation

Tumor cells can resist apoptosis, proliferate, and form malignant tumors [38]. Therefore, researching and intervening in apoptosis mechanisms is crucial for developing new anti-cancer treatment strategies. The influence of LINC01089 on cell death mechanisms is worth noting. In osteosarcoma, breast cancer, and lung adenocarcinoma cells, overexpression of LINC01089 can promote apoptosis and inhibit cell growth and proliferation [20, 23, 32]. Conversely, in HCC cells, LINC01089 knockdown hinders apoptosis and promotes malignant proliferation [25]. Interestingly, Li et al. [28] found that knocking down or overexpressing LINC01089 had no significant impact on apoptosis rates in non-small cell lung cancer cells, indicating that LINC01089-mediated promotion of NSCLC cell proliferation is due to cell cycle regulation rather than apoptosis. These findings collectively suggest that LINC01089 affects cell survival and proliferation through different mechanisms in various cancer types. Its potential as a therapeutic target is diverse and complex, warranting further in-depth research, though its role in regulating apoptosis and proliferation remains biologically significant.

### LINC01089 in tumor cell migration and EMT regulation

LINC01089 plays a crucial role in regulating tumor cell migration and epithelial-mesenchymal transition (EMT). EMT is vital in cancer progression, contributing significantly to the complex dynamics of tumorigenesis [39]. Changes in LINC01089 expression can markedly influence tumor cell migration and EMT processes. Functional biological analyses indicate that LINC01089 can inhibit lung cancer cell migration and invasion. Transwell and wound healing assays have shown that increasing LINC01089 expression in A549 and H-1975 cells inhibits cell migration [35]. Furthermore, a series of in vivo experiments confirmed the above functions of LINC01089 in LUAD cells [31]. Yang et al. [22] found that overexpression of LINC01089 in AGS and MNK-45 cells increased the expression of epithelial markers (E-cadherin) while decreasing mesenchymal markers (N-cadherin, Vimentin). Recent studies in small cell lung cancer (SCLC) revealed that LINC01089 knockdown mediated changes in the expression of N-cadherin, E-cadherin, Snail, Slug, and SRPR1 in A549 and H1299 cells, with protein levels of p-GSK-3β and β-catenin also increased following siLINC01089 transfection [28]. However, in HCC, LINC01089 exhibits a dual role. Su et al. [24] found that LINC01089 depletion in HCC cells reduced the expression of N-cadherin and Vimentin while increasing E-cadherin expression. Conversely, overexpression of LINC01089 increased N-cadherin and Vimentin expression while reducing E-cadherin expression. Additionally, SK-Hep-1 or HepG2 cells with LINC01089 knockdown showed significantly fewer pseudopodia, rounder shapes, lower length-to-width ratios, and fewer extremely elongated cells compared to control cells. This indicates that LINC01089 affects HCC cell morphology and migration ability by regulating EMT. Overall, LINC01089 is a significant contributor to tumor formation. However, its pan-cancer biological functions need further research. The role of LINC01089 in regulating tumor cell migration and EMT makes it a potential therapeutic target. Modulating LINC01089 expression could inhibit tumor migration and metastasis, improving treatment efficacy. Furthermore, LINC01089 expression levels could serve as biomarkers for assessing tumor invasiveness and metastasis risk, supporting clinical decision-making.

### LINC01089 inhibition of tumor cell chemoresistance

Chemoresistance remains a major obstacle in cancer treatment, significantly determining cancer mortality rates [40, 41]. EMT is an effective therapeutic target for cancer metastasis and chemoresistance [42]. Studies have shown that silencing LINC01089 can promote EMT and increase sorafenib resistance in HCC by upregulating miR-665 expression, thus enhancing invasion [25]. In addition, LINC01089 expression significantly affects chemotherapy sensitivity in SCLC cells. Downregulating LINC01089 markedly increased chemoresistance in chemotherapy-sensitive SCLC cells, while overexpression of LINC01089 increased chemotherapy sensitivity in chemoresistant SCLC cells. These results indicate that modulating LINC01089 expression can reverse chemoresistance in SCLC cells, enhancing the efficacy of chemotherapeutic drugs. Animal experiments further confirmed this, with tumor volumes in the LINC01089 knockdown group being significantly higher than those in the control group after chemotherapy, clearly illustrating LINC01089’s role in inhibiting tumor cell chemoresistance [33]. In summary, modulating LINC01089 expression can significantly affect tumor cell sensitivity and resistance to chemotherapeutic drugs. Overcoming LINC01089-mediated treatment resistance may bring new therapeutic hope to cancer patients. Research and development of interventions targeting LINC01089 could improve chemotherapy outcomes and patient prognosis.

## Mechanisms of LINC01089-mediated biological functions in different cancer

**Fig. 3:**
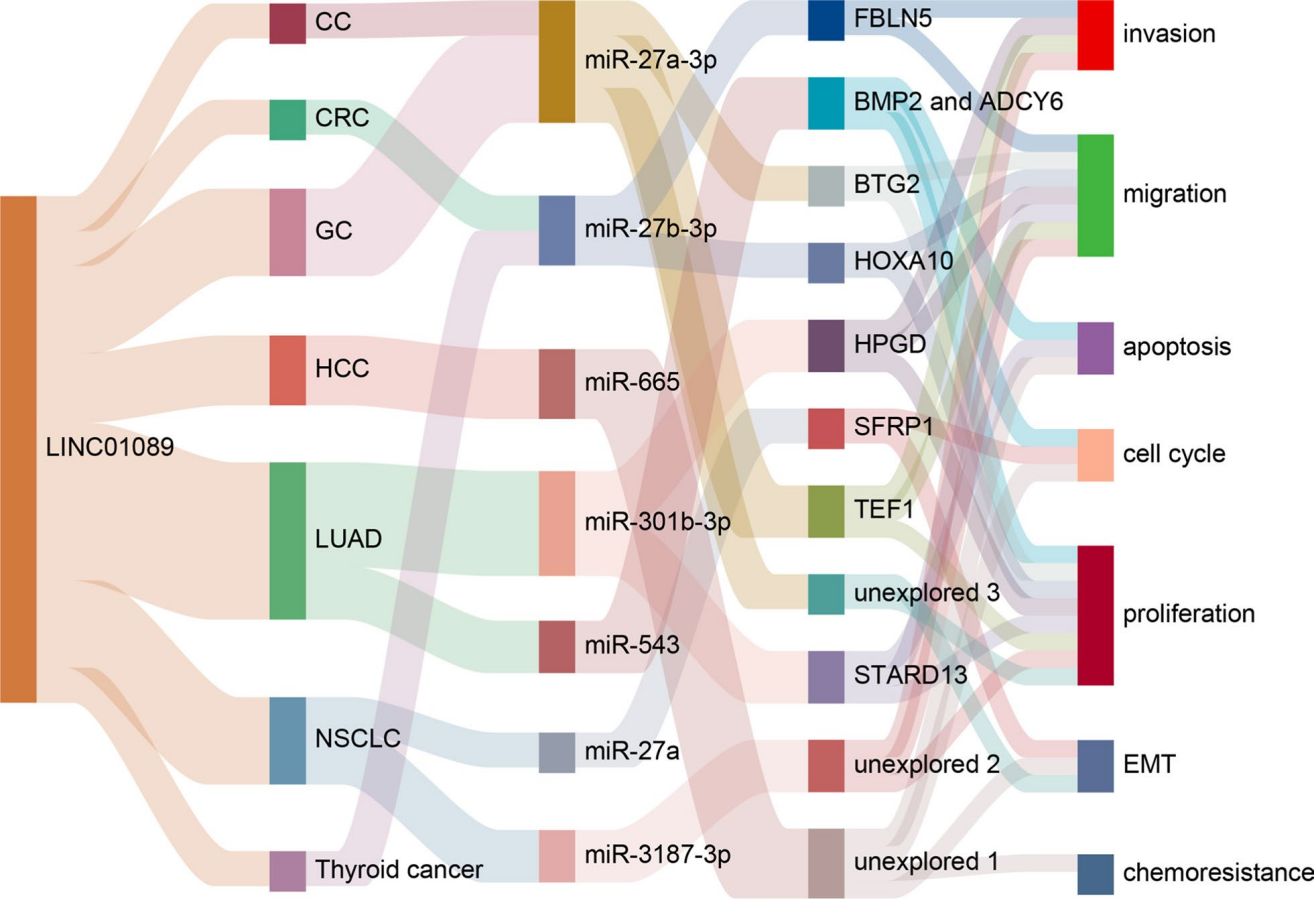
This figure demonstrates that LINC00518 sequesters miRNAs (miR-27a-3p, miR-27b-3p, miR-665, miR-301b-3p, miR-543, miR-27a, and miR-3187-3p) through the ceRNA network, thereby preventing them from binding to and regulating their target mRNAs. This modulation impacts various aspects of the cellular phenotype, including cell proliferation, migration, invasion, cell cycle, apoptosis, and chemoresistance

LINC01089 serves as a versatile regulator of gene expression, primarily operating through the ceRNA network. Its chief function is to sequester miRNAs, preventing them from binding to target mRNAs and thereby modulating their expression [43, 44] (as shown in Fig. 3). Additionally, LINC01089 plays a significant role in various tumor gene regulatory processes by acting as a modular scaffold, interacting with heterogeneous nuclear ribonucleoprotein M (hnRNPM), and influencing downstream molecular mediators. In terms of upstream mechanisms, the transcription of LINC00518 is driven not only by super-enhancers (SE) but also regulated by upstream molecular pathways, showcasing its intricate regulatory mechanisms in tumor biology.

### Hepatocellular carcinoma

**Fig. 4:**
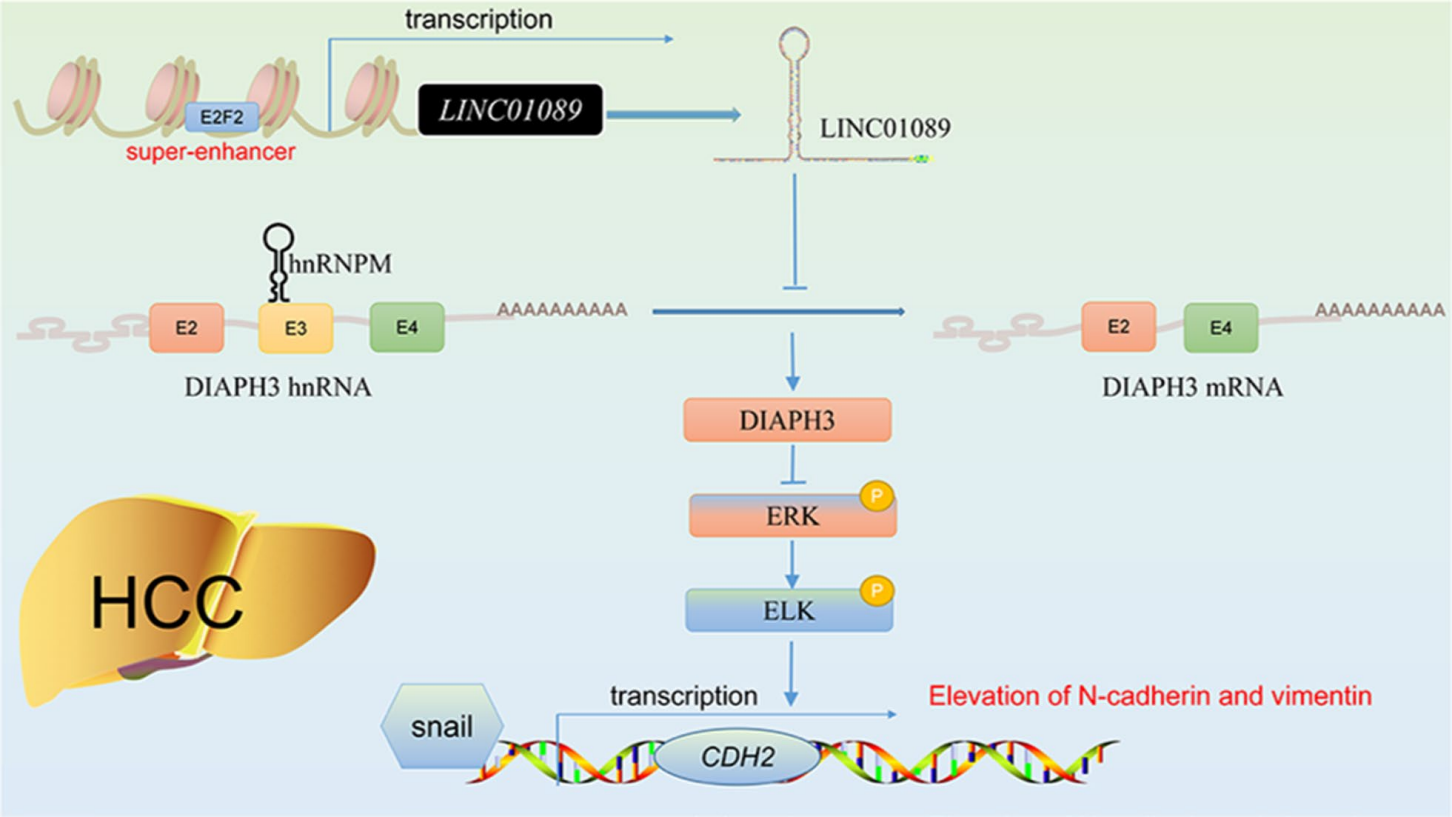
Regulation of LINC01089 gene expression as an oncogene in HCC. The E2F2 super-enhancer affects hnRNPM-mediated shearing by regulating the expression of LINC01089, which in turn alters the action of IGF2BP3 on m6A modification sites. This process leads to alterations in DIAPH3 mRNA transcription. This regulation further affects the levels of ERK and ELK proteins and upregulates the expression of N-calmodulin and vimentin via Snail, thereby contributing to the progression of HCC

Hepatocellular carcinoma (HCC) is the third leading cause of cancer-related mortality worldwide, with its death rate continuing to rise. As a prevalent form of primary liver cancer, both the incidence and mortality rates of HCC remain alarmingly high [45, 46]. Recent studies have highlighted the impact of aberrant LINC01089 expression on HCC progression. Evidence suggests that LINC01089 exhibits a dual role in HCC tumor tissues. On one hand, some studies report that LINC01089 is downregulated in HCC tissues, with its low expression correlating with larger tumor volumes, poorer TNM staging, and increased HBsAg levels. Conversely, other research indicates that LINC01089 is upregulated in HCC compared to normal liver tissue, with this upregulation enhancing HCC cell proliferation, metastasis, and invasion, while also inducing EMT. These findings suggest that LINC01089’s role in HCC may be complex, potentially acting both as a tumor suppressor and as a promoter of cancer progression under certain conditions. As a tumor suppressor, LINC01089 inhibits EMT in HCC cells by sequestering miR-665, thereby reducing resistance to sorafenib [25] (as shown in Fig. 3). Conversely, another study found that EGF can downregulate LINC01089 expression, leading to decreased E-cadherin and ZO-1 expression, and increased levels of vimentin [26]. As an oncogene, LINC01089’s transcription and overexpression are promoted by the binding of transcription factor E2F1 to its super-enhancer (SE). Subsequently, LINC01089 interacts with heterogeneous nuclear ribonucleoprotein M (hnRNPM), resulting in hnRNPM-mediated skipping of DIAPH3 exon 3, which diminishes IGF2BP3's recognition of m6A modification sites. This leads to increased DIAPH3 protein levels, thereby promoting the ERK/Elk1/Snail axis and enhancing EMT in HCC cells [24] (as shown in Fig. 4). In summary, LINC01089 exhibits a complex and dual role in HCC. A comprehensive understanding and evaluation of its biological functions, clinical significance, and potential risks and benefits in therapy are crucial for effectively utilizing LINC01089 as a biomarker and therapeutic target, thereby improving patient outcomes and prognosis.

### Non-small cell lung cancer

**Fig. 5:**
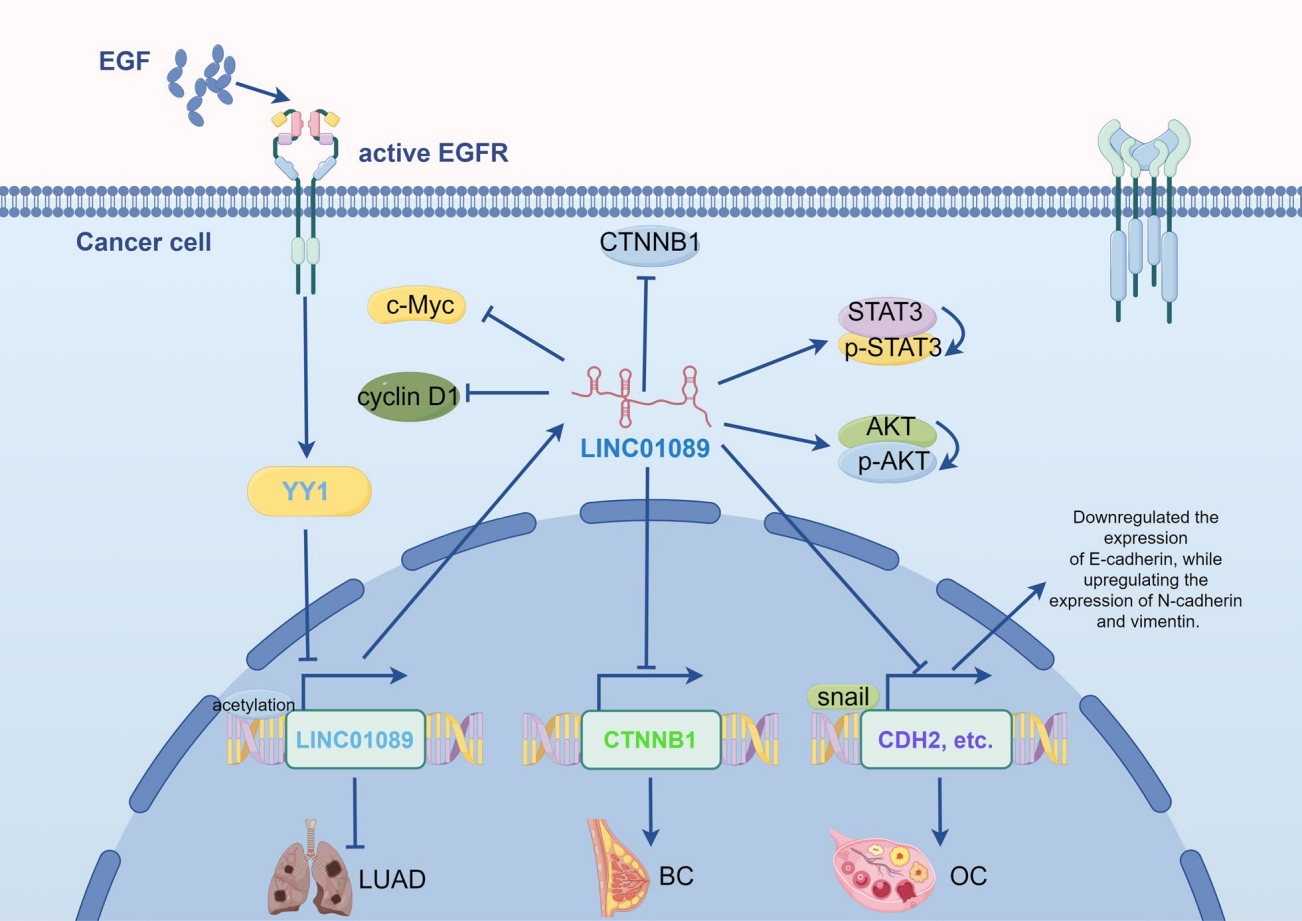
Following EGF treatment, YY1 transcriptionally represses LINC01089, thereby regulating CTNNB1, cyclin D1, P-STAT3, and P-AKT. This results in the downregulation of E-cadherin and upregulation of N-cadherin and vimentin, consequently promoting the progression of lung adenocarcinoma, breast cancer, and ovarian cance (by Figdraw2.0)

**Fig. 6:**
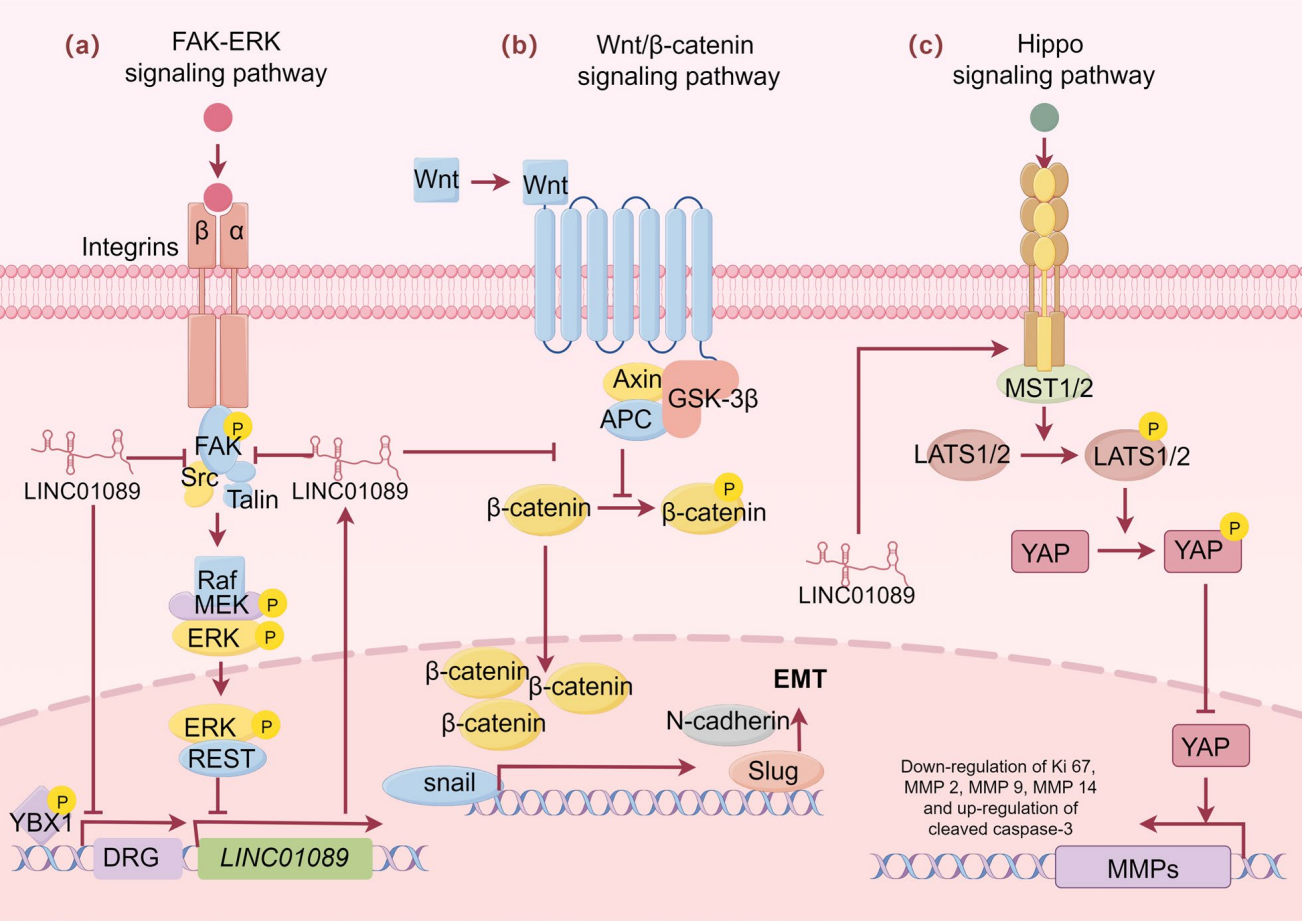
LINC01089 affects three key cell signaling pathways: FAK-ERK, Wnt/β-catenin, and Hippo. **a** The FAK-ERK pathway activates FAK and ERK through integrins, affecting cell proliferation and survival. **b** The Wnt/β-catenin pathway regulates the entry of β-catenin into the nucleus through Wnt ligands, impacting gene transcription. **c** The Hippo pathway regulates YAP activity through MST1/2 and LATS1/2, affecting the expression of genes related to cell proliferation (by Figdraw2.0)

Lung cancer remains one of the most prevalent cancers globally [1, 47], categorized primarily into non-small cell lung cancer (NSCLC) and small cell lung cancer (SCLC) [48, 49]. Due to the lack of discernible clinical symptoms, early diagnosis of lung cancer has been a significant challenge. Therefore, there is an urgent need for highly sensitive and specific diagnostic markers for lung cancer. Studies have shown that the expression of LINC01089 is significantly reduced in lung cancer tissues compared to adjacent normal tissues. The downregulation of LINC01089 promotes the proliferation, migration, and invasion of lung cancer cells [31]. Furthermore, reduced levels of LINC01089 are associated with larger tumor sizes and poorer histological differentiation in lung cancer patients [20]. These findings suggest that LINC01089 may serve as a potential diagnostic marker for lung cancer and is significant in prognostic evaluation. In the context of lung adenocarcinoma, overexpression of LINC01089 can counteract the promoting effects of miR-543 on tumor suppressor genes (BMP2 and ADCY6), inhibiting tumorigenesis and promoting apoptosis of tumor cells [20]. LINC01089 can also suppress the proliferation and migration of lung adenocarcinoma cells by regulating the miR-301b-3p/STARD13 axis [35]. Another study discovered that after EGF treatment, LINC01089 transcription can be directly inhibited by the transcriptional repressor YY1, which reduces the competitive endogenous RNA activity against miR-301b-3p, thereby decreasing HPGD expression and reducing levels of p-STAT3 and p-AKT [31] (as shown in Fig. 5). Additionally, LINC01089 mitigates tumor proliferation, migration, and invasion in A549 and SK-MES-1 cells by sponging miR-3187-3p [37]. Intriguingly, Wang et al. discovered that LINC01089 inhibits the development and progression of NSCLC by suppressing the miR-27a-SFRP1-Wnt/β-catenin-EMT pathway. Specifically, the overexpression of LINC01089 inhibits miR-27a through a sponging effect, thereby increasing SFRP1 expression, which in turn suppresses the Wnt/β-catenin signaling pathway. This suppression results in decreased mRNA levels of N-cadherin, Snail, and Slug, increased E-cadherin levels, and significantly reduced protein expression of p-GSK-3β and β-catenin in A549 cells, thereby attenuating the EMT process in NSCLC cells [28] (as shown in Fig. 6b). Overall, these findings underscore the critical role of LINC01089 in the early diagnosis, prognosis evaluation, and treatment of non-small cell lung cancer, indicating its potential as a biomarker and therapeutic target.

### Small cell lung cancer

Small cell lung cancer (SCLC) accounts for approximately 13% to 15% of all lung cancers and is characterized by high malignancy, invasiveness, and a propensity for metastasis [50, 51]. In SCLC research, Wang et al. [33] revealed the complex relationship between LINC01089, focal adhesion kinase (FAK), and the ERK signaling pathway. LINC01089 inhibits FAK activation by blocking its interaction with Src and Talin kinases. FAK activation recruits the REST transcription factor through the ERK signaling pathway, negatively regulating LINC01089 transcription. Knockdown of LINC01089 increases YBX1 phosphorylation levels, upregulates the expression of drug resistance-related genes, and promotes the formation of focal adhesions, indicated by markers such as Paxillin, FAK, and phalloidin staining. Moreover, EGF reactivates the FAK–ERK signaling pathway, suppressing LINC01089 expression. Phosphorylated ERK translocates to the nucleus, activating transcription factors and regulating the expression of related genes. The REST transcription factor regulates the transcriptional synthesis of LINC01089 without affecting its RNA degradation. A negative feedback loop exists between FAK and LINC01089, with their interaction dependent on the presence of a new FAK variant and the non-conserved region of LINC01089. During chemotherapy resistance, the upregulation of integrin β1 in SCLC cells further activates the FAK and ERK signaling pathways, leading to the suppression of LINC01089 expression. These findings highlight the critical role and regulatory mechanisms of LINC01089 in SCLC (as shown in Fig. 6a). Therefore, LINC01089 plays a pivotal role in SCLC through its complex regulatory mechanisms that intricately modulate the FAK–ERK signaling pathway. By regulating this pathway, LINC01089 significantly influences tumor invasiveness and chemoresistance, making it a promising therapeutic target in the treatment of SCLC.

### Breast cancer

Breast cancer (BC) is the second most common cancer globally and the leading cause of cancer-related death among women [52, 53]. The molecular mechanisms by which LINC01089 regulates breast cancer are diverse and complex. In the mRNA-lncRNA network model constructed by Dashti et al. [54], LINC01089 is identified as a crucial pathway and therapeutic target for breast cancer, introducing a precise method for discovering and prioritizing BC-related targets. Additionally, studies have found that EGF stimulation mediated by the RAS-to-ERK pathway leads to histone deacetylation near the transcription initiation site of LINC01089, rapidly downregulating its expression and promoting the migration and invasion of MCF10A cells [19]. Yuan et al. [23] discovered that LINC01089 inhibits the proliferation, migration, and invasion of BC cells by suppressing the transcription of β-catenin, thereby blocking the Wnt/β-catenin signaling pathway. Their research showed that in breast cancer cells overexpressing LINC01089, the levels of total β-catenin, active β-catenin (Ser45), active β-catenin (Ser33/Ser37/Thr41), and several downstream target proteins (including cyclin D1 and c-Myc) were significantly reduced. Additionally, the mRNA levels of β-catenin (CTNNB1) were also markedly downregulated, indicating that LINC01089’s inhibitory effect on β-catenin is primarily achieved through transcriptional regulation (as shown in Fig. 5). These findings further demonstrate the tumor-suppressive role of LINC01089 in breast cancer cells. However, current studies on LINC01089 in breast cancer are mostly indirect, and the elucidated mechanisms are not yet fully understood. Therefore, more foundational experiments are needed to deeply explore the specific mechanisms of LINC01089 in breast cancer.

### Gastric cancer

Gastric cancer (GC) is a primary epithelial malignancy originating in the stomach and ranks as the fifth leading cause of cancer-related deaths globally [1, 55–57]. Due to the lack of early symptoms and low rates of routine screening, most patients are diagnosed at advanced stages [58, 59]. Consequently, there is an urgent need to explore novel biomarkers and innovative therapeutic targets for early detection of GC. Research indicates that LINC01089 is consistently downregulated in both gastric cancer tissues and cell lines. The decreased levels of LINC01089 are significantly associated with larger tumor sizes, higher T staging, and lymph node metastasis [21]. Mechanistically, LINC01089 exerts its tumor-suppressive effects by competitively binding to miR-27a-3p, thereby promoting the expression of TET1 and inhibiting the malignant progression of breast cancer [21, 22]. TET1, a member of the TET family, induces DNA demethylation by converting 5-methylcytosine (5mC) to 5-hydroxymethylcytosine (5hmC), and is often downregulated in cancers [60, 61]. In studies examining Helicobacter pylori-induced gastric cancer, reduced expression of TET1 leads to decreased KLF4 levels and enhances the proliferation, migration, and colony formation of gastric epithelial and cancer cells[62]. Notably, TET1 is targeted by miR-27a-3p in gastric cancer and is positively regulated by LINC01089. Thus, the interaction between LINC01089 and TET1 plays a crucial role in regulating cancer progression. However, further research using animal models is needed to validate these findings and fully elucidate the role of LINC01089 in gastric cancer.

### Thyroid cancer

Thyroid cancer is the most common endocrine malignancy [63, 64]. Pan et al. [36] found that LINC01089 inhibits tumor progression in thyroid cancer by binding to miR-27b-3p and increasing the expression of FBLN5, which regulates cell proliferation, migration, and invasion. FBLN5 has also been shown to have tumor-suppressive effects in gastric cancer, breast cancer, and lung cancer [65–67]. Clinical investigations have revealed that lower LINC01089 expression correlates with higher tumor staging and regional lymph node metastasis, highlighting the significant potential of LINC01089 as a prognostic marker for thyroid cancer. However, the role of LINC01089 in thyroid cancer requires further validation in animal models, and given the limited number of thyroid cancer patients who experience metastasis and mortality, the relationship between LINC01089 expression and recurrence or death remains to be further explored.

### Ovarian cancer

In ovarian cancer, LINC01089 is downregulated by M2-like tumor-associated macrophages (TAMs) secreting EGF, which activates the EGFR-ERK signaling pathway. This downregulation of LINC01089 leads to reduced expression of E-cadherin and increased expression of N-cadherin and vimentin, promoting epithelial-to-mesenchymal transition (EMT) and resulting in poor prognosis [34]. Animal experiments have further demonstrated the critical role of LINC01089 in ovarian cancer tumorigenesis. Intraperitoneal injection of lentivirus encoding LINC01089 into a tumor-bearing nude mouse model significantly reduced the number and weight of tumor nodules, with tumor growth being markedly inhibited. These findings confirm that LINC01089 is an essential lncRNA in suppressing EGFR signaling-mediated ovarian cancer formation, suggesting that lncRNAs may provide more precise targets for TAM-targeted cancer therapies.

### Osteosarcoma

Osteosarcoma is the most common malignant tumor in childhood [68]. Zhang et al. [32] found that LINC01089 is downregulated in osteosarcoma cells compared to normal cells. YAP is a core effector molecule in the Hippo signaling pathway [69]. Upregulation of LINC01089 in U2 OS and Saos-2 cells led to a significant decrease in YAP protein levels and an increase in p-YAP levels, along with reduced Ki67, MMP2, MMP9, MMP14, and increased cleaved caspase-3, indicating suppression of cell proliferation, migration, and invasion, and promotion of apoptosis (as shown in Fig. 6c). Treatment with PY-60 (a YAP activator) elevated YAP levels while reducing p-YAP levels, counteracting the effects of LINC01089 overexpression by restoring YAP levels and negating the increase in YAP phosphorylation induced by LINC01089. This resulted in heightened malignant behavior of U2 OS cells, counteracting the suppressive effects of LINC01089 overexpression on cell proliferation, migration, and invasion, and diminishing the promotion of apoptosis by LINC01089 overexpression. Conversely, knockdown of LINC01089 produced opposite effects. Conversely, knockdown of LINC01089 resulted in opposite effects. These results suggest that LINC01089 suppresses osteosarcoma development via the Hippo pathway.

### Other malignant tumors

Apart from the specific cancers discussed earlier, LINC01089 is consistently downregulated in several other malignancies, including cervical cancer (CC), colorectal cancer (CRC) and glioma. This downregulation across various cancers suggests that LINC01089 may serve as an important biomarker and potential therapeutic target. Future research should delve deeper into the specific mechanisms by which LINC01089 functions in these cancers to elucidate its role in cancer progression and to advance related diagnostic and therapeutic strategies.

In colorectal cancer, LINC01089 can upregulate HOXA10 expression by sequestering miR-27b-3p, thereby inhibiting the proliferation and invasion of colorectal cancer cells [29]. HOXA10, a transcription factor, has been previously associated with the promotion of colorectal cancer development [70, 71]. These findings indicate that LINC01089 plays a critical tumor-suppressive role in colorectal cancer by regulating miR-27b-3p and its target genes. Similarly, in cervical cancer, LINC01089 also acts as a tumor suppressor by sponging miR-27a-3p to increase the expression of BTG2, thereby inhibiting cell proliferation and metastasis. Knockdown of LINC01089 has the opposite effect [27]. Recent studies suggest that BTG2 can block the Wnt/β-catenin signaling pathway, thus inhibiting cervical cancer cell growth [72]. This implies that LINC01089 might influence cervical cancer through miR-27a-3p and BTG2, potentially affecting the Wnt/β-catenin signaling pathway. However, direct experimental evidence is still lacking and further research is needed to validate this hypothesis. Furthermore, studies have shown that downregulation of LINC01089 expression is associated with poor prognosis in glioma patients [30]. Multivariate analysis revealed that decreased LINC01089 expression and high pathological grade for gliomas are independent predictors of poor patient prognosis. Additionally, glioma cells overexpressing LINC01089 exhibited reduced proliferation, migration, and invasion abilities in vitro, along with increased apoptosis, and suppressed tumor formation in nude mice in vivo. These findings suggest that LINC01089 may play a significant role in the onset and progression of glioma.

## Conclusion and future perspectives

Long non-coding RNAs (lncRNAs) have shown immense potential as emerging biomarkers in cancer research. They can be identified in various biological samples, including tissue, saliva, and plasma, and exhibit cell-specific or stage-specific expression patterns, making lncRNAs promising biomarkers. LINC01089 is a lncRNA that plays multiple roles in malignant tumors. Despite limited studies on liver cancer, LINC01089 is considered a tumor suppressor gene in almost all types of cancer. Research indicates that low levels of LINC01089 are associated with poorer clinical outcomes. LINC01089 functions through sponge effects on various miRNAs to regulate downstream targets, thereby influencing cancer progression. Additionally, LINC01089 regulates multiple mechanisms such as cell cycle, apoptosis, proliferation, migration, and invasion through pathways like FAK–ERK, Hippo, and Wnt/β-catenin, enhancing the invasiveness and metastatic potential of cancer cells. Notably, LINC01089 also reduces tumor cell chemoresistance by inhibiting EMT. These findings highlight the multifaceted roles of LINC01089 in cancer biology and its significant potential as a therapeutic target, providing new directions for future research and treatment strategies.

In terms of LINC01089-targeted therapies, current lncRNA targeting methods include RNA interference (RNAi), antisense oligonucleotides (ASO), CRISPR/Cas systems, small molecule inhibitors, LNA-modified oligonucleotides, GapmeRs, and overexpression systems [73]. For example, CRISPR-Cas13a platforms can be used for programmable knockdown of oncogenic lncRNAs, reducing off-target effects, while overexpression vectors can be utilized to promote the expression of tumor suppressor lncRNAs. Advances in these technologies provide a solid foundation for the potential of lncRNAs as biomarkers and therapeutic targets.

In this article, LINC01089 is predominantly characterized as a tumor suppressor gene. However, contradictory findings have emerged, with some studies suggesting a potential tumor-promoting role, underscoring the necessity for further rigorous investigations to elucidate the underlying mechanisms driving these divergent effects. Moreover, the therapeutic landscape for LINC01089 remains underexplored, with no targeted drugs currently available. Critical challenges such as drug-induced toxicity, off-target effects, and the development of safe and efficient delivery systems have yet to be addressed comprehensively. Finally, there is an urgent need to establish a standardized research framework encompassing robust detection methodologies and clinical application protocols, to fully harness LINC01089’s potential as a novel biomarker in oncology.

## Data Availability

Data sharing is not applicable to this article, as no data sets were generated or analyzed during the current study.

## Abbreviations

LINC01089: Long intergenic non-protein coding RNA 1089
lncRNA: Long non-coding RNA
ceRNA: Competing endogenous RNA
LIMT: Long non coding RNA inhibiting metastasis
EGF: Epidermal growth factor
SETD1B: SET domain containing 1B
RHOF: Ras homolog family member F
OS: Overall survival
PFS: Progression-free survival
ACC: Adrenocortical carcinoma
CESC: Cervical squamous cell carcinoma
BRCA: Breast cancer
UCEC: Uterine corpus endometrial carcinoma
LUSC: Lung squamous cell carcinoma
COAD: Colon adenocarcinoma
OV: Ovarian serous cystadenocarcinoma
ESCA: Esophageal carcinoma
KICH: Kidney chromophobe
LUAD: Lung adenocarcinoma
UCS: Uterine carcinosarcoma
THCA: Thyroid carcinoma
PRAD: Prostate adenocarcinoma
SKCM: Skin cutaneous melanoma
READ: Rectum adenocarcinoma
STAD: Stomach adenocarcinoma
CHOL: Cholangiocarcinoma
THYM: Thymoma
PCPG: Pheochromocytoma and paraganglioma
BLCA: Bladder urothelial carcinoma
HNSC: Head and neck squamous cell carcinoma
KIRP: Kidney renal papillary cell carcinoma
LGG: Lower grade glioma
PAAD: Pancreatic adenocarcinoma
KIRC: Kidney renal clear cell carcinoma
LIHC: Liver hepatocellular carcinoma
UVM: Uveal melanoma
EMT: Epithelial–mesenchymal transition
SRPR1: Signal recognition particle receptor subunit alpha
GSK-3β: Glycogen synthase kinase 3 beta
hnRNPM: Heterogeneous nuclear ribonucleoprotein M
SE: Super-enhancers
HBsAg: Hepatitis B surface antigen
ZO-1: Zonula occludens-1
E2F1: E2F transcription factor 1
DIAPH3: Diaphanous related formin 3
IGF2BP3: Insulin-like growth factor 2 mRNA-binding protein 3
ERK/Elk1: Extracellular signal-regulated kinase/ETS-like gene 1
BMP2: Bone morphogenetic protein 2
ADCY6: Adenylate cyclase 6
STARD13: StAR-related lipid transfer domain containing 13
YY1: Yin Yang 1 transcription factor
STAT3: Signal transducer and activator of transcription 3
AKT: Protein kinase B
CTNNB1: Catenin beta 1
ERK: Extracellular signal-regulated kinase
FAK: Focal adhesion kinase
REST: RE1-silencing transcription factor
5mC: 5-Methylcytosine
5hmC: 5-Hydroxymethylcytosine
FBLN5: Fibulin 5
HOXA10: Homeobox A10
BTG2: B-cell translocation gene 2
TAMs: Tumor-associated macrophages
YAP: Yes-associated protein
MMP2: Matrix metallopeptidase 2
MMP9: Matrix metallopeptidase 9
MMP14: Matrix metallopeptidase 14
RNAi: RNA interference
ASO: Antisense oligonucleotides
CRISPR/Cas: Clustered regularly interspaced short palindromic repeats/CRISPR-associated protein
